# Epidemiology, drug resistance, and clinical risk factors of peritoneal dialysis-associated peritonitis: a five-year multicenter study

**DOI:** 10.3389/fcimb.2025.1654246

**Published:** 2025-09-11

**Authors:** Min Zhang, Xiang Li, Yun Zhang, Jingxian Wu, Jie Liu, Yajuan Li, Anyong Wang, Yuanhong Xu, Bo Wang, Jinxing Xia

**Affiliations:** ^1^ Department of Clinical Laboratory, First Affiliated Hospital of Anhui Medical University, Hefei, China; ^2^ Department of Clinical Laboratory, Fuyang Hospital of Anhui Medical University, Fuyang, China; ^3^ Department of Clinical Laboratory, the Second Affiliated Hospital of Anhui University of Chinese Medicine, Hefei, China; ^4^ Department of Clinical Laboratory, Wulidun Street Community Health Service Center, Hefei, China; ^5^ Department of Clinical Laboratory, Dongcheng Branch of the First Affiliated Hospital of Anhui Medical University (Feidong People's Hospital), Hefei, China; ^6^ Department of Clinical Laboratory, Jiading Central Hospital of Shanghai, Shanghai, China

**Keywords:** peritoneal dialysis-associated peritonitis, microbiological surveillance, antimicrobial resistance, risk factors, *Staphylococcus, Candida*

## Abstract

**Background:**

Peritoneal dialysis-associated peritonitis (PDAP) remains a major complication in long-term dialysis patients, leading to significant morbidity and healthcare burden. This study aimed to investigate the microbial spectrum, antimicrobial resistance patterns, and clinical risk factors associated with PDAP in hospitalized patients in Anhui, China, over the past five years.

**Methods:**

A retrospective analysis was conducted on 438 peritoneal dialysis (PD) patients from three PD centers in Anhui from 2020 to early 2025. Of these, 238 patients were diagnosed with PDAP and 200 served as controls without peritonitis. Peritoneal effluents were cultured and microbiologically identified using MALDI-TOF MS and VITEK 2 systems. Antimicrobial susceptibility testing followed CLSI M100 standards. Clinical and laboratory data were statistically analyzed using SPSS v26.0, and multivariate logistic regression model was used to determine independent risk factors.

**Results:**

Significant differences were observed between the PDAP and control cohorts in sex, age, hospitalization time, PD duration, red blood cell count, total protein, albumin, blood glucose, and concomitant conditions (*e.g.*, hepatitis B, autoimmune diseases, and hyperthyroidism) (*p* < 0.05). Laboratory infectious markers including peripheral blood white blood cell (WBC) count, neutrophil percentage, procalcitonin (PCT), C-reactive protein, peritoneal dialysate WBC and multinucleated cell counts, were significantly elevated in the PDAP population compared to controls, with serum PCT and dialysate WBCs presented as significant predictors after multivariate adjustment. *Staphylococcus* species showed predominant methicillin resistance (47.22% oxacillin-susceptible) with moxifloxacin outperforming other fluoroquinolones, while carbapenems demonstrated near-universal efficacy against Enterobacterales (*esp.*, for ertapenem). *Candida* species mounted variable antifungal responses, with optimal activities of amphotericin B/flucytosine except fluconazole, underscoring both therapeutic opportunities and emerging resistance threats across bacterial and fungal pathogens.

**Conclusion:**

The multicenter study confirmed elevated serum PCT and peritoneal dialysate leukocytes as robust independent clinical predictors for PDAP, with other risk factors significantly increasing disease susceptibility. The diverse microbial spectrum and antimicrobial resistance features shed light on the importance of updated local microbial surveillance to guide empirical treatment and clinical management strategies on PDAP.

## Introduction

Peritoneal dialysis (PD) is an effective alternative therapy for patients with end-stage renal disease (ESRD), offering advantages such as home-based management, better preservation of residual kidney function, and cost-effectiveness compared to hemodialysis (National Kidney Foundation., 1997; Teitelbaum, 2021). However, peritoneal dialysis-associated peritonitis (PDAP) remains a critical infectious complication, leading to substantial morbidity, peritoneal membrane alterations, and increased healthcare burden (Brown et al., 2011; Hsieh et al., 2014a). Despite the availability of clinical guidelines from the International Society for Peritoneal Dialysis (ISPD), evident variability exists in the diagnosis and management of PDAP across global centers, with limited adherence to the recommended practices. Treatment failure rates range from 18.00% to 25.00%, and mortality associated with PDAP is estimated at approximately 3.50 - 10.00% (Mujais, 2006; Ghali et al., 2011; Cho and Johnson, 2014; Hsieh et al., 2014b; Tian et al., 2016; Nochaiwong et al., 2018; Salzer, 2018). Timely identification of causative pathogens and their antimicrobial resistance profiles is crucial for guiding empirical antibiotic therapies and optimizing patient outcomes.

The microbial profile of PDAP varies significantly across regions and institutions, reflecting differences in local antimicrobial stewardship and microbial ecology. While Gram-positive bacteria (*e.g.*, *Staphylococcus* and *Streptococcus* species) remain predominant pathogens, rising trends in Gram-negative bacterial and fungal infections have been documented (Camargo et al., 2021; Yin et al., 2022; Liu et al., 2023; Freitas and Calice Silva, 2024). Furthermore, the extensive use of broad-spectrum antibiotics and prophylactic empirical treatments has driven dynamic shifts in both pathogen distribution and antibiotic resistance patterns within PD centers. Although international guidelines provide general recommendations, region-specific epidemiological data are critical for optimizing targeted antimicrobial strategies in PD patients.

Additionally, the incidence and severity of PDAP are strongly associated with multiple risk factors, including advanced age, poor nutritional status, prolonged dialysis duration, elevated inflammatory markers, *etc.* Clinical studies indicate that PDAP contributes to increased hospitalization rates, peritoneal function impairment, and residual kidney function deterioration, all of which remarkably impact patients’ quality of life. Notably, PDAP is the leading cause of temporary or permanent discontinuation of PD therapy (Szeto and Li, 2019). Therefore, comprehensive investigations into PDAP pathogen profiles, resistance patterns as well as clinical risk factors are essential for developing effective prevention strategies and guiding clinical antimicrobial applications in PDAP management.

This study was conducted in collaboration with three PD centers in Anhui Province, involving a multicenter retrospective analysis of clinical data from 438 hospitalized PD patients over a 5-year period. It systematically examined the distribution patterns of pathogens causing PDAP, temporal trends in antimicrobial susceptibility, and key clinical and laboratory indicators related to infection risks and patient prognosis. The aim was to establish a dynamically updated microbiological guidance framework to support the diagnosis and treatment of PDAP in the local areas. The findings also offered region-specific evidence to inform broader strategies for the prevention and management of PD-related infections globally.

## Materials and methods

### Study design and clinical data collection

This multicenter retrospective study was conducted at three PD centers in Anhui Province, China. Hospitalized patients were screened undergoing PD between January 2020 and February 2025. A total of 438 patients were enrolled and stratified into two groups: 238 patients diagnosed with peritoneal dialysis-associated peritonitis (PDAP group) and 200 patients receiving regular PD without peritonitis (non-PDAP group).

According to the ISPD 2022 guidelines, PDAP can be diagnosed based on at least two of the three criteria: 1) clinical features consistent with peritonitis, *i.e.*, abdominal pain and/or cloudy dialysis effluent; 2) peripheral blood white blood cell (WBC) count > 100/µL or > 0.1 × 10^9^/L (after a dwell time of at least 2 h), with > 50% polymorphonuclear leukocytes; and 3) positive dialysis effluent cultures (Yin et al., 2022). The inclusion criteria were as follows: 1) aged 18 - 80 years; 2) diagnosed with uremia and have received continuous PD treatment for ≥ 3 months prior to enrollment (Li et al., 2022). The exclusion criteria were: 1) incomplete clinical records or follow-up data; 2) concurrent participation in other interventional clinical trials; 3) recovery of residual kidney function (with subsequent discontinuation of PD therapy); 4) presence of hemorrhagic or chylous peritoneal effluent; 5) neuropsychiatric disorders affecting treatment compliance; 6) comorbid active hematologic diseases (acute/chronic); 7) history of cerebrovascular events (*e.g.*, cerebral infarction or hemorrhage); 8) active severe infectious diseases with transmission risks; or 9) failure to complete follow-up per protocol or premature withdrawal from treatment.

The subject medical profiles and laboratory testing data were accessed through the hospital digitized retrieval systems. A variety of potential risk factors were comprehensively assessed including age, sex, occupation, duration of PD, anemia level (evaluated via red blood cells or RBCs, and hemoglobin), nutritional status (total protein and serum albumin), inflammatory markers (procalcitonin or PCT, C-reactive protein or CRP, peripheral blood WBC, dialysate WBC, and dialysate multinucleated cells), comorbidities (*e.g.*, hypertension, diabetes, heart diseases, hepatitis B, autoimmune diseases, and hyperthyroidism), prognosis, hospitalization duration, and total hospitalization costs. All the laboratory tests were performed in the certified facilities using standardized analyzers including Roche Cobas 8000 (Roche Diagnostics GmbH, Germany), Sysmex XN series (Sysmex Corporation, Japan), and Getein1600 (GeteinBiotech, China).

### Sample collection and organism identification

Peritoneal fluid samples were collected at the time of patient admission and subsequently delivered for routine laboratory tests and bacterial cultures. Samples were processed within 2 h of collection and subject to both aerobic and anaerobic cultures using BACTEC FX (Becton Dickinson, USA) or BacT/ALERT systems (bioMérieux, France), depending on center availability. Positive cultures were further subcultured onto appropriate media (blood agar, MacConkey, and Sabouraud dextrose agar) and incubated at 35°C for 48 h or more. The organism identification was via a VITEK mass spectrometry platform using a matrix-assisted laser desorption ionization-time of flight mass spectrometry (MALDI-TOF MS, bioMérieux, France) according to the manufacturer’s instructions. The quality control bacterium was involved with *Escherichia coli* (ATCC 8739).

### Antimicrobial susceptibility testing

AST was performed through the method of broth micro-dilution or automated VITEK 2 system, with interpretations based on CLSI M100 performance standards (Institute, C.a.L.S., 2024). For Gram-positive bacteria, the antimicrobials were tested including penicillins (penicillin G and oxacillin), macrolides (erythromycin), tetracyclines and derivatives (tigecycline and tetracycline), fluoroquinolones (levofloxacin, ciprofloxacin and moxifloxacin), lincosamides (clindamycin), sulfonamide combinations (trimethoprim-sulfamethoxazole), aminoglycosides (gentamicin), rifamycins (rifampin), streptogramins (quinupristin/dalfopristin), oxazolidinones (linezolid), glycopeptides (vancomycin); for Gram-negative bacteria, antimicrobials tested were involved with penicillins (penicillin G and ampicillin), β-lactamase inhibitor combinations (ampicillin/sulbactam, piperacillin/tazobactam, cefoperazone/sulbactam, and ceftazidime/avibactam), cephalosporins (cefazolin, cefuroxime, ceftazidime, cefotaxime, ceftriaxone and cefepime), carbapenems (imipenem, meropenem and ertapenem), monobactams (aztreonam), aminoglycosides (gentamicin, tobramycin and amikacin), fluoroquinolones (ciprofloxacin and levofloxacin), sulfonamide combinations (trimethoprim-sulfamethoxazole), tetracyclines and derivatives (tigecycline and tetracycline); and antifungal drugs (amphotericin B, 5-fluorocytosine, and clinically commonly-used azoles). The quality control bacteria included *Escherichia coli* (ATCC 25923), *Staphylococcus aureus* (ATCC 25923), and *Pseudomonas aeruginosa* (ATCC 27853).

### Statistical analysis

Data were analyzed using SPSS software (version 26.0; IBM Corp., USA). Continuous variables were expressed as mean ± standard deviation (SD) or median with interquartile range (IQR) as appropriate, and compared using Student’s *t*-test or Mann-Whitney *U* test. Multivariate logistic regression was performed to identify independent risk factors for PDAP, with inclusion criteria set at *p* < 0.05 in univariate analysis. A two-tailed test with *p* < 0.05 was considered statistically significant.

## Results

### Baseline characteristics of the study population

**Figure 1:**
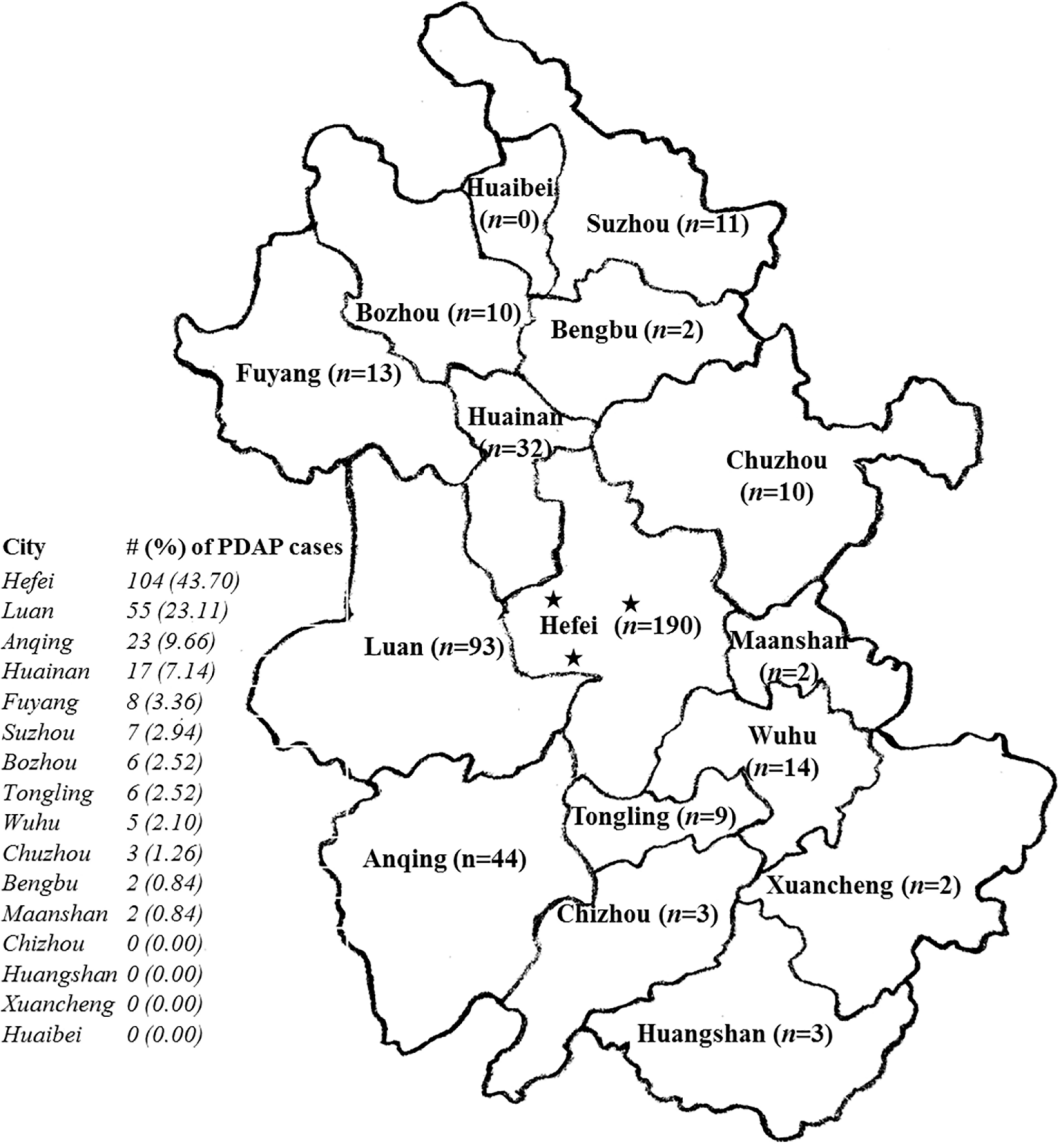
The geographical distribution of the recruited patients with peritoneal dialysis-associated peritonitis (PDAP) in the study. The five-pointed stars in the map indicated the locations of the 3 peritoneal dialysis (PD) centers within the provincial capital city of Hefei with a wide distribution of patients radiating across nearly the entire province; n, the number of PD patients recruited locally in the map; the # (%) of PDAP cases meant the number (#) of PDAP patients involved in each indicated city, as well as its percentage (%) accounted for among all the PDAP population of the study.

From January 2020 through February 2025, totally 438 PD patients were recruited from three PD centers affiliated to the First Affiliated Hospital of Anhui Medical University, the largest comprehensive Class A tertiary hospital with more than 6,000 beds in Anhui Province. Among them, 238 subjects were diagnosed with PDAP, and the rest were controls without peritonitis. All the PD centers are located in Hefei, the capital city of the province, with a wide distribution of patients radiating across nearly the entire province. In terms of the geographical distribution of PDAP subjects, Hefei presented the highest proportion of PDAP population (43.70%, n = 104), followed by other local regions including Luan (23.11%), Anqing (9.66%), Huainan (7.14%), *etc.* (**Figure 1**).

Statistically significant differences were observed between the PDAP and non-PDAP groups in terms of sex, age, PD duration, RBC count, total protein, albumin, blood glucose, and the presence of concomitant diseases such as hepatitis B virus infections, autoimmune diseases, and hyperthyroidism (*p* < 0.05 for all the comparisons). Patients in the PDAP group experienced longer hospital stays, incurred higher hospitalization costs, and had poorer clinical outcomes compared to the control cohort (*p* < 0.05). No significant differences were found between them pertaining to the occupation, certain underlying conditions, hemoglobin (Hb) levels, or platelet (PLT) counts. However, the laboratory markers of infection, including peripheral blood WBC count, neutrophil percentage, PCT, CRP, peritoneal dialysate WBC count, and the presence of multinucleated cells in dialysate, were significantly elevated in the PDAP group (all *p* < 0.001). Detailed comparisons of clinical and demographic variables are shown in **Tables 1**, **2**. To be noted, there was a relatively significant trend toward a worse prognosis or outcome for patients with PADP-associated fungal infections (**Supplementary Table S1**). Subsequent multivariate logistic regression analysis identified elevated serum PCT levels (OR 1.414, 95% CI 1.070 - 1.869) and increased peritoneal dialysate WBC counts (OR 1.082, 95% CI 1.033 - 1.133) as independent risk factors for the occurrence of PDAP in PD patients (*p* < 0.05), while CRP and peripheral blood WBC counts were not retained as significant predictors in the final model of analysis (**Table 3**).

**Table 1:** Demographic and clinical baseline of all the recruited PD patients in the study.

Factors	Variables	PDAP group n = 238, (%)	non-PDAP group n = 200, (%)	*χ^2^*	*p*
Sex	*Male*	126 (52.90)	80 (40.00)	7.31	0.01
	*Female*	112 (47.10)	120 (60.00)		
Age (years)	*0 - 19*	2 (0.80)	1 (0.50)	34.84	< 0.001
	*20 - 44*	33 (13.90)	64 (32.00)		
	*45 - 59*	108 (45.40)	99 (49.50)		
	*≥ 60*	95 (39.90)	36 (18.00)		
Occupation	*Workers*	18 (7.60)	15 (7.50)	2.58	0.76
	*Officials*	1 (0.40)	0 (0.00)		
	*Farmers*	67 (28.20)	49 (24.50)		
	*Retirees*	12 (5.00)	13 (6.50)		
	*Unemployed*	38 (16.00)	39 (19.50)		
	*Self-employed*	102 (42.90)	84 (42.00)		
Hospitalization duration (days)	*< 14*	126 (52.90)	175 (87.50)	60.38	< 0.001
	*≥ 14*	112 (47.10)	25 (12.50)		
Hospitalization cost (CNY)	*< 6,000*	3 (1.30)	91 (45.50)	232.50	< 0.001
	*6,000 - 12,000*	29 (12.20)	78 (39.00)		
	*> 12,000*	206 (86.60)	31 (15.50)		
Hypertension	*Yes*	85 (35.70)	84 (42.00)	1.81	0.18
	*No*	153 (64.30)	116 (58.00)		
Diabetes	*Yes*	29 (12.20)	19 (9.50)	0.80	0.37
	*No*	209 (87.80)	181 (90.50)		
Heart diseases	*Yes*	25 (10.50)	28 (14.00)	1.25	0.26
	*No*	213 (89.50)	172 (86.00)		
Hepatitis B	*Yes*	28 (11.80)	12 (6.00)	4.35	0.04
	*No*	210 (88.20)	188 (94.00)		
Autoimmune diseases	*Yes*	10 (4.20)	2 (1.00)	4.18	0.04
	*No*	228 (95.80)	198 (99.00)		
Hyperthyroidism	*Yes*	6 (2.50)	0 (0.00)	5.11	0.02
	*No*	232 (97.50)	200 (100.00)		
Prognosis	*Improvement*	228 (95.80)	200 (100.00)	8.60	< 0.001
	*Deterioration*	10 (4.20)	0 (0.00)		

PD, peritoneal dialysis; PDAP, peritoneal dialysis-associated peritonitis; non-PDAP group, PD patients without peritonitis; CNY, Chinese Yuan.

**Table 2:** Clinical and laboratory characteristics of the PD patients with or without PDAP.

Factors	PDAP group n = 238, (%)	non-PDAP group n = 200, (%)	*t/z*	*p*
PD duration (years)	3.10 (1.40-5.90)	2.15 (0.50-5.40)	-2.93	< 0.001
Neutrophil %	80.99 ± 11.80	70.04 ± 8.81	-11.03	< 0.001
RBC (× 10^12^/L)	3.24 ± 0.69	3.07 ± 0.69	-2.54	0.01
Hb (g/L)	97.00 (84.00-111.50)	94.50 (80.00-108.75)	-1.59	0.11
PLT (× 10^9^/L)	191.61 ± 68.34	182.82 ± 63.40	-1.38	0.17
TP (g/L)	59.53 ± 7.59	63.07 ± 7.78	4.79	< 0.001
ALB (g/L)	31.74 ± 5.29	35.27 ± 5.71	6.67	< 0.001
WBC (× 10^9^/L)	7.8 (5.83-10.89)	6.30 (4.97-7.86)	-5.89	< 0.001
PCT (ng/mL)	1.68 (0.47-10.86)	0.19 (0.10-0.30)	-13.12	< 0.001
CRP (mg/L)	52.67 (16.34-107.66)	1.67 (0.68-5.68)	-14.21	< 0.001
Dialysate WBC (× 10^6^/L)	2,325 (628.00-5,310.50)	3.00 (1.00-6.00)	-17.58	< 0.001
Dialysate multinucleated cells (× 10^6^/L)	2,077 (445.00-4,618.00)	1.00 (0.00-2.00)	-17.39	< 0.001
eGFR	5.00 (4.00-6.00)	4.00 (4.00-5.00)	-3.57	< 0.001
GLU (mmol/L)	6.83 (5.81-7.73)	6.32 (5.39-7.30)	-2.61	0.01

PD, peritoneal dialysis; PDAP, peritoneal dialysis-associated peritonitis; non-PDAP group, PD patients without peritonitis; RBC, red blood cell; Hb, hemoglobin; PLT, platelet; TP, total protein; ALB, albumin; WBC, white blood cell; PCT, procalcitonin; CRP, C-reactive protein; eGFR, estimated glomerular filtration rate; GLU, blood glucose.

**Table 3:** Multivariate logistic regression analysis for infectious risk factors.

Variables	*p*	OR	95% CI
WBC (× 10^9^/L)	0.993	1.001	0.794-1.261
PCT (ng/mL)	0.015	1.414	1.070-1.869
CRP (mg/L)	0.123	1.019	0.995-1.044
Dialysate WBC (× 10^6^/L)	0.001	1.082	1.033-1.133

WBC, white blood cell; PCT, procalcitonin; CRP, C-reactive protein.

### Microbiological profile of PDAP

Among the 238 PDAP cases, Gram-positive cocci were the predominant pathogens, accounting for 68.91% of all isolates, with *Staphylococcus* spp. and *Streptococcus* spp. being the most frequently identified. Gram-negative bacilli (mainly Enterobacteriaceae) comprised 20.59% of all isolates. And fungal agents (primarily *Candida* spp.) accounted for 4.20% of all isolates.

Among the Gram-positive organisms (n = 179) isolated from PDAP cases, *Staphylococcus* spp. were the most prevalent, accounting for 60.34% (n = 108), their most common species was *Staphylococcus epidermidis* (43.52%, n = 47), followed by *Staphylococcus aureus* (14.81%, n = 16), *Staphylococcus capitis* (11.11%, n = 12), *Staphylococcus haemolyticus* (10.19%, n = 11), and others. *Streptococcus* spp. were isolated in 51 cases, with *Streptococcus oralis* (39.22%, n = 20) and *Streptococcus salivarius* (21.57%, n = 11) being the most common species, followed by *Streptococcus mitis* (n = 5) and others. The Gram-positive bacteria also involved *Enterococcus* spp. (n = 5), *Corynebacterium* spp. (n = 5), *Bacillus* spp. (n = 2), *Kocuria* spp. (n = 2), and certain rare bacteria. Among the Gram-negative isolates (n = 49), *Escherichia coli* was the most frequently detected agent (44.90%, n = 22), followed by *Klebsiella* spp. (n = 6), *Enterobacter cloacae* (n = 2), *Salmonella* sp. (n = 1), *Hafnia alvei* (n = 1), *Neisseria* spp. (n = 5), *Moraxella osloensis* (n = 3), *Acinetobacter* spp. (n = 3), *Pseudomonas aeruginosa* (n = 3), and other rare ones. Fungal infections (n = 10) were primarily caused by *Candida* spp. (90.00%, n = 9), with *Candida parapsilosis* (44.44%, n = 4) and *Candida glabrata* (22.22%, n = 2) being relatively the most frequently isolated species. Other *Candida* species and *Trichosporon* sp. were also identified (**Table 4**).

**Table 4:** Microbiological profile from the patients with PDAP in the study.

Pathogens	# (%) of cases	Pathogens	# (%) of cases
**Gram-positive bacteria**		**Gram-negative bacteria**	
***Staphylococcus* spp.**	***108 (45.38%)***	***Escherichia* spp.** *(Escherichia coli)*	***22 (9.24%)***
*Staphylococcus epidermidis*	47 (43.52%)	***Klebsiella* spp.**	***6 (2.52%)***
*Staphylococcus aureus*	16 (14.81%)	*Klebsiella aerogenes*	3 (50.00%)
*Staphylococcus capitis*	12 (11.11%)	*Klebsiella pneumoniae*	2 (33.33%)
*Staphylococcus haemolyticus*	11 (10.19%)	*Klebsiella oxytoca*	1 (16.67%)
*Staphylococcus hominis*	8 (7.41%)	***Enterobacter* spp.** *(Enterobacter cloacae)*	***2 (0.84%)***
*Staphylococcus warneri*	3 (2.78%)	***Salmonella* sp.**	***1 (0.42%)***
*Other Staphylococcus* spp.	11 (10.19%)	***Hafnia* sp.** *(Hafnia alvei)*	***1 (0.42%)***
***Streptococcus* spp.**	***51 (21.43%)***	***Neisseria* spp.**	***5 (2.10%)***
*Streptococcus oralis*	20 (39.22%)	*Neisseria sicca*	2 (40.00%)
*Streptococcus salivarius*	11 (21.57%)	*Neisseria subflava*	1 (20.00%)
*Streptococcus mitis*	5 (9.80%)	*Neisseria mucosa*	1 (20.00%)
*Streptococcus agalactiae*	3 (5.89%)	*Neisseria elongata ss. glycolytica*	1 (20.00%)
*Streptococcus vestibularis*	3 (5.89%)	***Moraxella* spp.** *(Moraxella osloensis)*	***3 (1.26%)***
*Other Streptococcus* spp.	9 (17.65%)	***Acinetobacter* spp.**	***3 (1.26%)***
***Enterococcus* spp.**	***5 (2.10%)***	*Acinetobacter baumannii*	2 (66.67%)
*Enterococcus faecalis*	3 (60.00%)	*Acinetobacter* sp.	1 (33.33%)
*Enterococcus faecium*	2 (40.00%)	***Pseudomonas* spp.** *(Pseudomonas aeruginosa)*	***3 (1.26%)***
***Corynebacterium* spp.**	***5 (2.10%)***	***Other Gram-negative bacteria^#^***	***3 (1.26%)***
*Corynebacterium amycolatum*	3 (60.00%)	**Fungi**	
*Corynebacterium striatum*	1 (20.00%)	***Candida* spp.**	***9 (3.78%)***
*Other Corynebacterium* sp.	1 (20.00%)	*Candida parapsilosis*	4 (44.44%)
***Bacillus* spp.** *(Bacillus cereus, Bacillus pumilus)*	***2 (0.84%)***	*Candida glabrata*	2 (22.22%)
***Kocuria* spp.** *(Kocuria kristinae, Kocuria rosea)*	***2 (0.84%)***	*Other Candida* spp.*^¶^*	3 (33.33%)
Other Gram-positive bacteria^*^	***6 (2.52%)***	***Trichosporon* sp.**	***1 (0.42%)***

*, Other Gram-positive bacteria included Microbacterium sp., Rothia sp., Tsukamurella sp., Gemella sanguinis, Actinomyces turicensis and Abiotrophia defectiva. #, Other Gram-negative bacteria were Chryseomonas luteola, Paracoccus yeei and Bacteroides vulgatus. ¶, Other Candida spp. included Candida albicans, Candida guilliermondii and Candida tropicalis.

The bold values meant the total number (percentage) of the indicated microorganism genus or the given group.

To investigate the dynamic epidemiological trends over the 5-year study period, we stratified the analysis of pathogen distribution by year. The overall distribution of major pathogen groups (Gram-positive and Gram-negative bacteria, and fungi) remained relatively stable from 2020 to 2024, without statistically significant trend observed (*χ^2^* = 3.672, *p* = 0.903). Gram-positive bacteria were the predominant agents each year, ranging from 68.57% to 81.25% (**Supplementary Table S2**). Detailed pathogen distribution information is shown in **Supplementary Table S3**.

### Antimicrobial resistance profiles

The analysis revealed that 47.22% of *Staphylococcus* spp. were susceptible to oxacillin, confirming the predominance of methicillin-resistant strains. Moxifloxacin emerged as the most effective fluoroquinolone (12.04% resistant, MIC50/90: 0.25/2.00 μg/mL), outperforming ciprofloxacin (30.56%) and levofloxacin (34.26%). Although clindamycin and tetracycline demonstrated high susceptibility (85.98% and 77.78%, respectively), tetracycline resistance (21.30%) remained a concern. Intermediate resistance was observed for trimethoprim-sulfamethoxazole and gentamicin (67.60% and 82.41% susceptible, respectively), suggesting cautious clinical application (**Table 5**). To dynamically evaluate antimicrobial resistance trends for *Staphylococcus* spp. over the 5-year study period, a year-by-year analysis was further performed. Resistance to penicillin G remained consistently high (> 90.00% annually). Resistance to oxacillin showed a considerable fluctuation, with rates of 39.39% in 2020, peaking at 75.00% in 2022, and 70.00% in 2024. Similarly, their resistance rates to trimethoprim-sulfamethoxazole also varied, peaking at 43.75% in 2022 before decreasing. Importantly, no resistance was detected for vancomycin, linezolid, and tigecycline throughout the entire 5-year period (**Supplementary Table S4**).

**Table 5:** Antimicrobial features of the microorganisms from the PDAP patients.

Antimicrobial agents	R (%)	I (%)	S (%)	MIC50	MIC90
Staphylococcus spp.					
Penicillin G	93.52	0.00	6.48	0.50	8.00
Oxacillin	52.78	0.00	47.22	4.00	4.00
Erythromycin	60.00	0.00	40.00	8.00	8.00
Tetracycline	21.30	0.93	77.78	1.00	16.00
Tigecycline	0.00	0.00	100.00	0.13	0.25
Levofloxacin	34.26	2.78	62.96	0.50	8.00
Ciprofloxacin	30.56	6.48	62.96	1.00	8.00
Moxifloxacin	12.04	25.93	62.04	0.25	2.00
Clindamycin	14.02	0.00	85.98	0.25	8.00
TMP-SMX	32.41	0.00	67.60	12.00	192.00
Gentamicin	9.26	8.33	82.41	0.50	8.00
Rifampin	1.85	0.00	98.15	0.50	1.00
Quinupristin/dalfopristin	0.93	0.00	99.07	0.25	0.50
Linezolid	0.00	0.00	100.00	2.00	2.00
Vancomycin	0.00	0.00	100.00	1.00	2.00
Streptococcus spp.					
Penicillin G	0.00	33.33	66.67	0.13	1.00
Ampicillin	0.00	22.22	77.78	0.25	0.50
Cefotaxime	9.68	12.90	77.42	/	/
Ceftriaxone	11.43	5.70	82.90	0.13	32.00
Erythromycin	52.78	30.56	16.67	/	/
Levofloxacin	20.80	4.16	75.00	2.00	8.00
Clindamycin	36.59	4.88	58.54	0.25	32.00
Chloramphenicol	10.00	10.00	80.00	/	/
Tetracycline	40.00	25.00	35.00	16.00	16.00
Tigecycline	0.00	0.00	100.00	0.13	0.13
Quinupristin/dalfopristin	0.00	0.00	100.00	0.25	1.00
Linezolid	0.00	0.00	100.00	2.00	2.00
Vancomycin	0.00	0.00	100.00	0.50	1.00
All Enterobacterales					
Ampicillin	52.00	4.00	44.00	32.00	32.00
Piperacillin	42.86	0.00	57.14	/	/
Ampicillin/sulbactam	29.17	20.90	50.00	4.00	32.00
Piperacillin/tazobactam^*^	6.67	/	90.00	4.00	8.00
Cefoperazone/sulbactam	4.00	4.00	92.00	/	/
Cefazolin	42.50	0.00	57.50	4.00	64.00
Ceftazidime	16.67	0.00	83.30	1.00	16.00
Cefotaxime	22.22	0.00	77.78	/	/
Ceftriaxone	25.00	0.00	75.00	1.00	64.00
Cefuroxime	26.32	15.79	57.89	/	/
Cefepime^*^	16.67	/	73.33	1.00	16.00
Aztreonam	25.00	3.57	71.43	1.00	64.00
Gentamicin	34.62	0.00	65.38	1.00	16.00
Tobramycin	33.33	0.00	66.67	1.00	8.00
Amikacin	3.45	0.00	96.55	2.00	2.00
Imipenem	3.33	3.33	93.33	1.00	1.00
Ertapenem	0.00	0.00	100.00	0.50	0.50
Meropenem	5.00	0.00	95.00	/	/
Levofloxacin	34.48	31.03	34.48	1.00	8.00
Ciprofloxacin	40.74	3.70	55.56	0.25	4.00
TMP-SMX	46.67	0.00	53.33	24.00	384.00
Minocycline	0.00	5.56	94.44	/	/
Tigecycline	0.00	0.00	100.00	/	/
Ceftazidime/avibactam	0.00	0.00	100.00	/	/

*, SDD (%) of piperacillin/tazobactam and cefepime were 3.33% and 10.00%, respectively. SDD, susceptible-dose dependent; MIC, minimal inhibitory concentration; MIC50 or MIC90, MIC at which 50.00% or 90.00% of the isolates tested were inhibited; TMP-SMX, trimethoprim-sulfamethoxazole.

None of *Streptococcus* spp. isolates exhibited full resistance to penicillin G and ampicillin, though intermediate susceptibility was spotted (33.33% and 22.22%, respectively). Ceftriaxone maintained superior third-generation cephalosporin activity (82.90% susceptible, MIC50/90: 0.13/32.00 μg/mL), albeit with 11.43% of resistance. Alarmingly, erythromycin and tetracycline resistance affected 83.33% and 65.00% of isolates, respectively. While clindamycin resistance reached 36.59% (MIC90: 32.00 μg/mL). And levofloxacin and chloramphenicol remained moderately potent (75.00% and 80.00% susceptible, respectively). For Enterobacterales, carbapenems demonstrated exceptional efficacies: ertapenem (100.00% susceptible, MIC50/90: 0.50/0.50 μg/mL), imipenem (93.33%), and meropenem (95.00%). On the contrary, ampicillin, trimethoprim-sulfamethoxazole, and ciprofloxacin showed high resistance rates (52.00%, 46.67% and 40.74%, respectively). β-lactamase inhibitor combinations displayed divergent performances: the susceptibility to piperacillin/tazobactam (90.00%) and cefoperazone/sulbactam (92.00%) significantly surpassed ampicillin/sulbactam (50.00%). The third-generation cephalosporins presented moderate susceptibility (ceftazidime, 83.30%; cefotaxime, 77.78%), though elevated MIC90 values (ceftriaxone, 64.00 μg/mL) indicated emerging resistance (**Table 5**).

Antifungal testing against *Candida* spp. revealed that amphotericin B and flucytosine were the most potent (MIC50/90: 0.50/1.00 μg/mL and 4.00/4.00 μg/mL, respectively). Fluconazole showed bimodal activity (57.14% susceptible, MIC50: 1.00 μg/mL *vs.* MIC90: 32.00 μg/mL), while voriconazole maintained low MIC values (MIC50/90: 0.06/0.13 μg/mL) with 62.50% of susceptibility. Itraconazole demonstrated strong *in vitro* efficacy (MIC50/90: 0.13/0.50 μg/mL), in contrast to the resistant subset of fluconazole. Due to their limited clinical breakpoints (CBPs), these antifungal testing data were for exploratory purposes only (**Supplementary Table S5**).

## Discussion

The multicenter retrospective study, conducted over a five-year period in Anhui Province, comprehensively characterized the clinical features, risk factors, microbial spectrum, and antimicrobial susceptibility patterns of PDAP. The findings provided updated insights into the evolving epidemiology and management challenges of PDAP in the regional cohort.

The patients with PDAP exhibited featured demographic and clinical profiles, aligning with and extending the existing knowledge of associated risk factors in the present study. The PDAP cohort tended significantly to be older and had a longer PD duration, and the comorbidities such as hepatitis B, autoimmune diseases, and hyperthyroidism were more prevalent, underscoring that advanced age, prolonged dialysis-related exposure, and immune dysregulation were crucial contributors to peritonitis susceptibility (Nochaiwong et al., 2018; Yin et al., 2022; You et al., 2024). The increased susceptibility in older adults was likely multifactorial, attributable to immunosenescence (the age-related decline in immune function) as well as increased frailty and a higher cumulative burden of comorbidities, which collectively impaired host defenses against infections (Xia et al., 2022). Additionally, this study may suggest a potential sex disparity in PDAP incidence, a finding that warrants further exploration. Potential hypotheses for such a difference could include hormonal influences, as estrogen has been suggested to have immunomodulatory effects, or potential variations in hygiene practices and adherence to sterile techniques between sexes (Sciarra et al., 2023). However, it is also important to consider potential sample bias. The demographic profile observed in our cohort might reflect the specific epidemiology of end-stage renal disease in Anhui Province rather than a universal biological predisposition. Therefore, these demographic associations should be interpreted with caution and warrant validation in diverse populations (Ballinger et al., 2014). Furthermore, significant differences in non-infectious laboratory parameters, including RBC count, total protein, albumin, and blood glucose levels, were observed between PDAP and non-PDAP groups, suggesting PDAP impact on host metabolic states. Indeed, hyperglycemia and hypoproteinemia were reported as established risk factors for peritonitis in PD patients (Contreras-Velazquez et al., 2008; Wang et al., 2020). It was also recognized that uremia combined with diabetes led to more pronounced peritoneal membrane alterations than isolated uremia, and individuals on PD were subject to chronic nutritional deficiencies and compromised immunity, increasing their vulnerability to microbial colonization and peritonitis. Post-peritonitis, altered peritoneal vascular permeability was able to exacerbate nutritional deterioration by enhancing protein loss into the dialysate (Contreras-Velazquez et al., 2008; Wang et al., 2020). Therefore, targeted nutritional interventions would be exceptionally pivotal. For inflammatory markers, PDAP patients mounted significantly elevated systemic and local inflammatory responses, including WBC counts, neutrophil percentages, CRP, PCT, peritoneal dialysate WBC counts, and polymorphonuclear cell proportions. While CRP elevation was a known associated risk factor (Hind et al., 1985; Troidle et al., 2005), its diagnostic specificity was still limited (Yin et al., 2022). In contrast, PCT’s rapid elevation kinetics post-bacterial endotoxin exposure and non-reactivity to viral or sterile inflammation established its superior discriminative capacity (Shen et al., 2013; Yu et al., 2013; Yang et al., 2014). Notably, in our study, only PCT and dialysate WBC count remained significant predictors of PDAP after multivariate adjustment, suggesting their utility as more reliable early diagnostic indicators.

Accurate microbiological identification is fundamental for guiding PDAP management, as emphasized by current guidelines (Li et al., 2022). Our analysis of the microbial spectrum revealed that Gram-positive cocci were the predominant pathogens, constituting more than one-third of all cultured isolates. Within this group, *Staphylococcus* spp., and specifically *S. epidermidis*, were the most frequently isolated, a pattern consistent with previous epidemiological reports (Alwakeel et al., 2011; Trinh et al., 2019; You et al., 2024). The high prevalence of *S. epidermidis* likely pointed to cutaneous contamination and breaches in aseptic technique during PD procedures, highlighting a critical need for enhanced patient education on sterile protocols. Gram-negative organisms also represented a significant portion of the microbial landscape, with Enterobacteriaceae comprising over half of these isolates. Fungal peritonitis, although less common, was identified, with *Candida* species being the primary fungal pathogens. Given its association with severe outcomes, including the need for catheter removal (Juarez Villa et al., 2022), fungal PDAP remains a critical concern.

The antimicrobial resistance patterns observed in this study poses ongoing challenges for empirical therapy and requirements of robust stewardship. Among Gram-positive isolates, a high prevalence of methicillin-resistant *Staphylococcus* strains (MRSA and MRSE) was noted, together with poor susceptibility to penicillin and erythromycin. These findings support the use of vancomycin or linezolid for suspected Gram-positive infections, aligning with current ISPD guidelines (Li et al., 2022). A temporal analysis was further conducted to address potential epidemiological and antimicrobial shifts over the five-year period. Our findings suggest that while the overall distribution of major pathogen groups would remain stable, the resistance patterns of key pathogens such as *Staphylococcus* spp. could exhibit certain annual fluctuations, particularly for oxacillin. This variation highlights the dynamic nature of antimicrobial resistance and reinforces the need for ongoing surveillance. The consistently preserved susceptibility of *Staphylococcus* spp. to vancomycin, linezolid, and tigecycline was a crucial observation, providing confidence in their continued use as first-line or empirical agents for severe Gram-positive infections in the local areas. For Gram-negative organisms, particularly Enterobacteriaceae, though high sensitivity to carbapenems and tetracyclines was observed, there was an increasing trend of resistance to third-generation cephalosporins. This shift likely reflects selective pressure from broad-spectrum antibiotic use and reinforces the necessity for dedicated antimicrobial stewardship programs in long-term dialysis patients (Kitterer et al., 2015; Cho and Struijk, 2017; Zeng et al., 2021). Encouragingly, no carbapenem-resistant Enterobacteriaceae (CRE) were detected, suggesting preserved efficacy of these last-line agents in our setting.

In the context of fungal infections, approximately 40.00% of *Candida* isolates demonstrated reduced susceptibility to azoles. This necessitates clinical awareness, particularly for patients with prior antifungal exposure or prolonged antibiotic therapy. Echinocandins or amphotericin B should be considered as alternative treatments in cases of suspected or confirmed azole resistance. Our findings were consistent with studies showing a high rate of treatment failure for PDAP-associated fungal infections, which usually required catheter removal. It underscores the importance of early species identification and tailoring antifungal strategies (Li et al., 2022). Continuous surveillance of local resistance patterns is crucial for optimizing empirical treatment strategies and preserving the effectiveness of available antimicrobials.

## Conclusion

The five-year multicenter investigation provided comprehensive epidemiological and clinical profiles of PDAP. It revealed that elevated serum PCT and dialysate WBC count were reliable independent diagnostic predictors for PDAP. Advanced age, prolonged PD duration, and specific comorbidities significantly increased disease susceptibility. The diverse microbial spectrum and antimicrobial resistance features substantially complicated the empirical treatment. The findings reinforced the urgent need to tailor antimicrobial therapy to local epidemiological and clinical characteristics, and highlighted the necessity of optimized catheter care, early risk stratification, and antimicrobial stewardship programs, to improve clinical outcomes in the PD population.

## Data Availability

The original contributions presented in the study are included in the article/**Supplementary Material**. Further inquiries can be directed to the corresponding authors.
