# Supplementary material for: Epidemiology, drug resistance, and clinical risk factors of peritoneal dialysis-associated peritonitis: a five-year multicenter study
Source: Front Cell Infect Microbiol. 2025 Sep 11;15:1654246. doi: 10.3389/fcimb.2025.1654246 (PMC12460227; doi:10.3389/fcimb.2025.1654246)
Supplement: Supplementary file 1 [file DataSheet1.pdf]

Supplementary Materials

Table S1. Clinical outcomes of PDAP-associated fungal infections.

Subjects	Sex	Age (years)	Pathogens	Prognosis/Outcomes*
P1	F	52	*Trichosporon* sp.	Cured
P2	M	56	*Candida parapsilosis*	Improved
P3	F	62	*Candida parapsilosis*	Improved
P4	F	70	*Candida parapsilosis*	Improved
P5	F	54	*Candida tropicalis*	Improved
P6	F	61	*Candida glabrata*	Improved
P7	F	36	*Candida glabrata*	Deteriorated
P8	F	53	*Candida albicans*	Deteriorated
P9	F	57	*Candida parapsilosis*	Deteriorated
P10	F	67	*Candida guilliermondii*	Moribund

Notes: *, All the subjects neither received catheter removal nor switched to hemodialysis treatment throughout the entire therapeutic courses, and the deteriorated outcomes included those discharged with voluntary withdrawal of treatment. P, patient; F, female; M, male.

Table S2. Detailed microbiological profiles of PDAP patients over the 5-year study period.

Group	2020 [n (%)]	2021 [n (%)]	2022 [n (%)]	2023 [n (%)]	2024 [n (%)]	χ^2	*p*
Gram-positive bacteria	52 (81.25%)	35 (74.47%)	24 (68.57%)	40 (75.47%)	28 (71.79%)		
Gram-negative bacteria	10 (15.63%)	10 (21.28%)	10 (28.57%)	10 (18.87%)	9 (23.08%)	3.672	0.903
Fungi	2 (3.12%)	2 (4.25%)	1 (2.86%)	3 (5.66%)	2 (5.13%)		

Table S3. Detailed pathogen distribution information of PDAP patients in the study.

Pathogens	# (%) of cases					
	Total	2020	2021	2022	2023	2024
**Gram-positive bacteria**						
Staphylococcus spp.	***108 (45.38%)***	***33***	***21***	***16***	***28***	***10***
Staphylococcus epidermidis	47 (43.52%)	12	9	6	12	8
Staphylococcus aureus	16 (14.81%)	8	2	2	3	1
Staphylococcus capitis	12 (11.11%)	5	2	2	3	0
Staphylococcus haemolyticus	11 (10.19%)	3	4	1	3	0
Staphylococcus hominis	8 (7.41%)	3	1	2	2	0
Staphylococcus warneri	3 (2.78%)	1	1	1	0	0
Other Staphylococcus spp.	11 (10.19%)	1	2	2	5	1
Streptococcus spp.	***51 (21.43%)***	***14***	***10***	***5***	***7***	***15***
Streptococcus oralis	20 (39.22%)	7	4	3	4	2
Streptococcus salivarius	11 (21.57%)	2	2	0	1	6
Streptococcus mitis	5 (9.80%)	0	1	1	1	2
Streptococcus agalactiae	3 (5.89%)	2	1	0	0	0
Streptococcus vestibularis	3 (5.89%)	0	0	0	1	2
Other Streptococcus spp.	9 (17.65%)	3	2	1	0	3
Enterococcus spp.	***5 (2.10%)***	***0***	***1***	***2***	***2***	***0***
Enterococcus faecalis	3 (60.00%)	0	1	1	1	0
Enterococcus faecium	2 (40.00%)	0	0	1	1	0
Corynebacterium spp.	***5 (2.10%)***	***2***	***1***	***0***	***1***	***1***
Corynebacterium amycolatum	3 (60.00%)	1	1	0	1	0
Corynebacterium striatum	1 (20.00%)	1	0	0	0	0
Other Corynebacterium sp.	1 (20.00%)	0	0	0	0	1
Bacillus spp. *(Bacillus cereus, Bacillus pumilus)*	***2 (0.84%)***	***1***	***0***	***0***	***0***	***1***
Kocuria spp. *(Kocuria kristinae, Kocuria rosea)*	***2 (0.84%)***	***0***	***1***	***0***	***0***	***1***
Other Gram-positive bacteria	***6 (2.52%)***	***2***	***1***	***1***	***2***	***0***
**Gram-negative bacteria**						
Escherichia spp. *(Escherichia coli)*	***22 (9.24%)***	***2***	***6***	***5***	***5***	***4***
Klebsiella spp.	***6 (2.52%)***	***0***	***2***	***0***	***2***	***2***
Klebsiella aerogenes	3 (50.00%)	0	1	0	0	2
Klebsiella pneumoniae	2 (33.33%)	0	0	0	2	0
Klebsiella oxytoca	1 (16.67%)	0	1	0	0	0
Enterobacter spp. *(Enterobacter cloacae)*	***2 (0.84%)***	***0***	***0***	***0***	***1***	***1***
Salmonella sp.	***1 (0.42%)***	***0***	***1***	***0***	***0***	***0***
Hafnia sp. *(Hafnia alvei)*	***1 (0.42%)***	***0***	***0***	***1***	***0***	***0***
Neisseria spp.	***5 (2.10%)***	***2***	***1***	***1***	***1***	***0***
Neisseria sicca	2 (40.00%)	1	0	1	0	0
Neisseria subflava	1 (20.00%)	0	1	0	0	0
Neisseria mucosa	1 (20.00%)	0	0	0	1	0

Neisseria elongata ss. glycolytica	1 (20.00%)	1	0	0	0	0
Moraxella spp. *(Moraxella osloensis)*	***3 (1.26%)***	***2***	0	1	0	0
Acinetobacter spp.	***3 (1.26%)***	***1***	0	1	0	1
Acinetobacter baumannii	2 (66.67%)	0	0	1	0	1
Acinetobacter sp.	1 (33.33%)	1	0	0	0	0
Pseudomonas spp. *(Pseudomonas aeruginosa)*	***3 (1.26%)***	1	0	1	1	0
Other Gram-negative bacteria	***3 (1.26%)***	***2***	0	***0***	***0***	***1***
**Fungi**						
Candida spp.	***9 (3.78%)***	***2***	***2***	***1***	***2***	***2***
Candida parapsilosis	4 (44.44%)	1	0	0	2	1
Candida glabrata	2 (22.22%)	1	0	1	0	0
Other Candida spp.	3 (33.33%)	0	2	0	0	1
Trichosporon sp.	***1 (0.42%)***	***0***	***0***	***0***	***1***	***0***

Table S4. Detailed antimicrobial profiles of *Staphylococcus* species from PDAP patients.

Antimicrobial agents	Resistance (%) of *Staphylococcus* spp.					
	**Total (n = 108)**	**2020 (n = 33)**	**2021 (n = 21)**	**2022 (n = 16)**	**2023 (n = 28)**	**2024 (n = 10)**
Penicillin G	93.52	90.91	90.48	100.00	92.86	100.00
Oxacillin	52.78	39.39	57.14	75.00	46.43	70.00
Erythromycin*	60.00	53.85	/	/	/	/
Tetracycline	21.30	12.12	19.05	25.00	28.57	30.00
Tigecycline	0.00	0.00	0.00	0.00	0.00	0.00
Levofloxacin	34.26	33.33	47.62	43.75	21.43	30.00
Ciprofloxacin	30.56	33.33	42.86	31.25	17.86	30.00
Moxifloxacin	12.04	15.15	19.05	12.50	3.57	10.00
Clindamycin	14.02	12.12	14.29	18.75	14.81	10.00
TMP-SMX	32.41	27.27	38.10	43.75	28.57	30.00
Gentamicin	9.26	9.09	14.29	12.50	7.14	0.00
Rifampin	1.85	3.03	4.76	0.00	0.00	0.00
Quinupristin/dalfopristin	0.93	0.00	4.76	0.00	0.00	0.00
Linezolid	0.00	0.00	0.00	0.00	0.00	0.00
Vancomycin	0.00	0.00	0.00	0.00	0.00	0.00

Notes: *, "/" indicated the sample size of < 10 cases each year. TMP-SMX, trimethoprim-sulfamethoxazole.

Table S5. Antimicrobial features of *Candida* species from PDAP patients.

***Candida* spp.**	**R (%)**	**I (%)**	**S (%)**	**SDD (%)***	**MIC50**	**MIC90**
Amphotericin B	/	/	/	100.00	0.50	1.00
Flucytosine	/	/	/	100.00	4.00	4.00
Fluconazole	14.29	28.57	57.14	/	1.00	32.00
Itraconazole	/	/	/	100.00	0.13	0.50
Voriconazole	/	/	62.50	37.50	0.06	0.13

Notes: *, No CLSI-approved breakpoint is available for those *Candida* spp. R, resistant; I, intermediate; S, susceptible or sensitive; SDD, susceptible-dose dependent; MIC, minimal inhibitory concentration; MIC50 or MIC90, MIC at which 50.00% or 90.00% of the isolates tested were inhibit.
